# Supplementary material for: Predicting visual function by interpreting a neuronal wiring diagram
Source: Nature. 2024 Oct 2;634(8032):113–23. doi: 10.1038/s41586-024-07953-5 (PMC11446822; doi:10.1038/s41586-024-07953-5)
Supplement: Supplementary file 6 — CRF and ERF predictions for individual TmY4 and TmY9 cells. Analogous to Supplementary Data 3, but for TmY target types. Shown are the top four monosynaptic pathways, the strongest pathway passing through each of the top ten intermediary types (ranking from Extended Data Fig. 7), and the trisynaptic pathway Tm1–TmY–Dm3–TmY (see the section entitled Prediction of spatial normalization). [file 41586_2024_7953_MOESM6_ESM.zip › DataS4/TmY9qΓèÑ/720575940628437269.pdf]

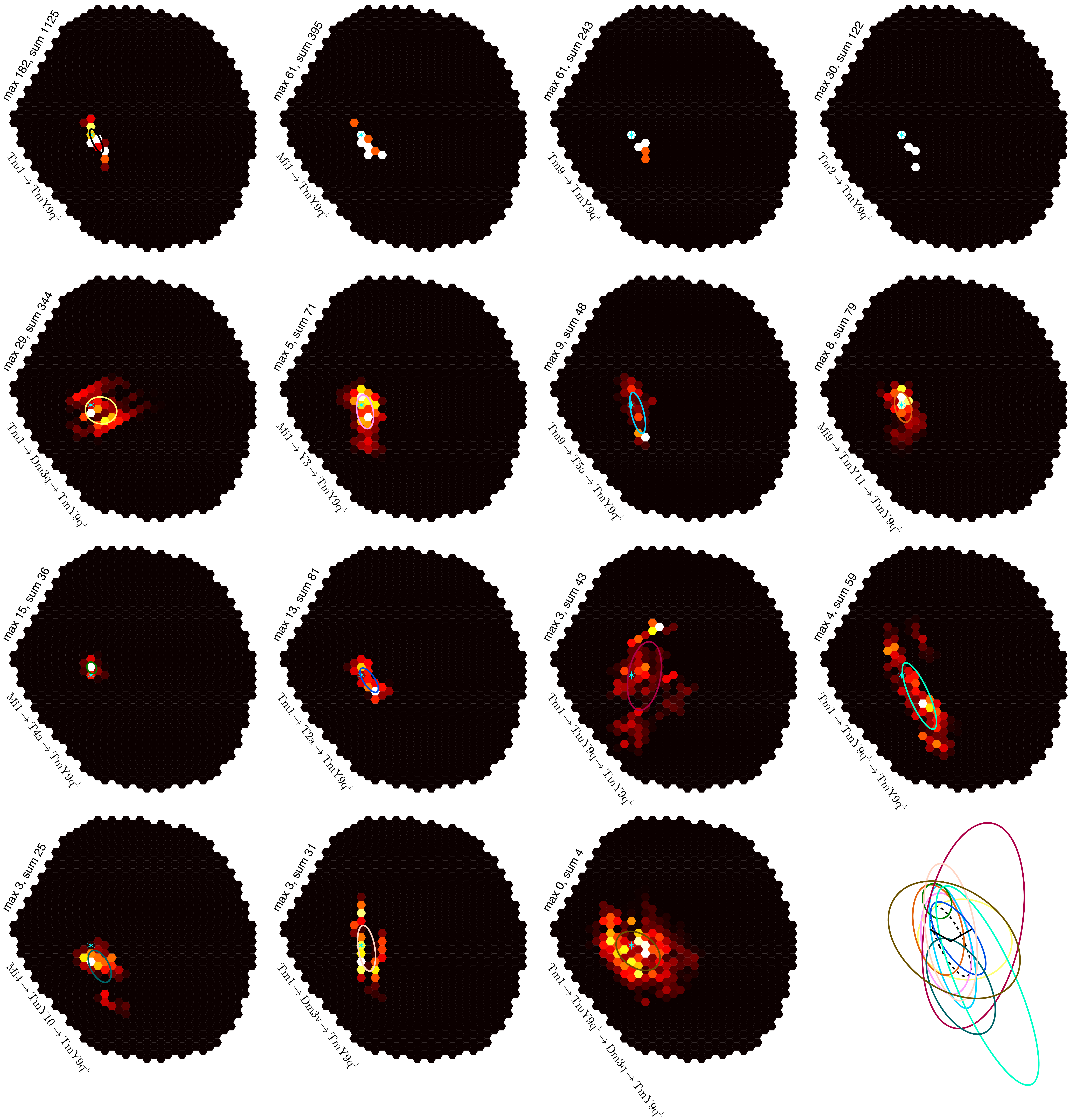

max 182, sum 1125
Tm1 → TmY9q⊥
max 61, sum 395
Mi1 → TmY9q⊥
max 61, sum 243
Tm9 → TmY9q⊥
max 30, sum 122
Tm2 → TmY9q⊥
max 29, sum 344
Tm1 → Dm3q → TmY9q⊥
max 5, sum 71
Mi1 → Y3 → TmY9q⊥
max 9, sum 48
Tm9 → T5a → TmY9q⊥
max 8, sum 79
Mi9 → TmY11 → TmY9q⊥
max 15, sum 36
Mi1 → T4a → TmY9q⊥
max 13, sum 81
Tm1 → T2a → TmY9q⊥
max 3, sum 43
Tm1 → TmY9q → TmY9q⊥
max 4, sum 59
Tm1 → TmY9q⊥ → TmY9q⊥
max 3, sum 25
Mi4 → TmY10 → TmY9q⊥
max 3, sum 31
Tm1 → Dm3v → TmY9q⊥
max 0, sum 4
Tm1 → TmY9q⊥ → Dm3q → TmY9q⊥
